# Cardiac Adiposity and Arrhythmias: The Role of Imaging

**DOI:** 10.3390/diagnostics11020362

**Published:** 2021-02-20

**Authors:** Maria Bonou, Sophie Mavrogeni, Chris J. Kapelios, George Markousis-Mavrogenis, Constantina Aggeli, Evangelos Cholongitas, Athanase D. Protogerou, John Barbetseas

**Affiliations:** 1 Department of Cardiology, Laiko General Hospital, 11527 Athens, Greece; bonou.maria@yahoo.com (M.B.); jbnv@otenet.gr (J.B.); 2 Department of Cardiology, Onassis Cardiac Surgery Center, 17674 Athens, Greece; sophie.mavrogeni@gmail.com (S.M.); georgemm32@gmail.com (G.M.-M.); 3 First Department of Cardiology, Hippokration General Hospital, Medical School of National & Kapodistrian University, 11527 Athens, Greece; dina.aggeli@gmail.com; 4 First Department of Internal Medicine, Medical School of National & Kapodistrian University, 11527 Athens, Greece; cholongitas@yahoo.gr; 5 Cardiovascular Prevention & Research Unit, Clinic and Laboratory of Pathophysiology, National & Kapodistrian University Athens School of Medicine, 11527 Athens, Greece; aprotog@med.uoa.gr

**Keywords:** adipose tissue, cardiac fat, arrhythmogenesis, atrial fibrillation, cardiac magnetic resonance, echocardiography

## Abstract

Increased cardiac fat depots are metabolically active tissues that have a pronounced pro-inflammatory nature. Increasing evidence supports a potential role of cardiac adiposity as a determinant of the substrate of atrial fibrillation and ventricular arrhythmias. The underlying mechanism appears to be multifactorial with local inflammation, fibrosis, adipocyte infiltration, electrical remodeling, autonomic nervous system modulation, oxidative stress and gene expression playing interrelating roles. Current imaging modalities, such as echocardiography, computed tomography and cardiac magnetic resonance, have provided valuable insight into the relationship between cardiac adiposity and arrhythmogenesis, in order to better understand the pathophysiology and improve risk prediction of the patients, over the presence of obesity and traditional risk factors. However, at present, given the insufficient data for the additive value of imaging biomarkers on commonly used risk algorithms, the use of different screening modalities currently is indicated for personalized risk stratification and prognostication in this setting.

This review focuses on the diagnostic imaging approaches in the evaluation of cardiac adiposity and their efficacy to predict future arrhythmological risk along with discussion of underlying disease mechanisms, based on current available evidence.

## 1. Cardiac Adiposity Pathophysiology 

Overwhelming evidence supports the idea that adipose tissue acts as an endocrine organ having a significant impact on cardiovascular function [1]. Obesity is associated with adipose tissue dysfunction including increased proinflammatory and decreased anti-inflammatory factors secretion, thus contributing to insulin resistance, glucose intolerance, hypertension and abnormal lipid metabolism that are often seen in obese people [1,2]. These alterations affect the heart and vessels resulting in an increase in cardiovascular (CV) events. This risk is significantly linked to the distribution of fat rather than body mass index (BMI) or total adiposity, being much higher in the presence of visceral adipose tissue (VAT) and increased ectopic fat accumulation in normally lean organs, such as the liver, heart and skeletal muscles [2,3,4].

Increased cardiac fat depots are metabolically active tissues having a pronounced pro-inflammatory nature, which is enhanced in obesity and type-2 diabetes [3]. Ectopic cardiac fat may be located pericardially (the adipose tissue surrounds the parietal pericardium), epicardially (adipose tissue between the myocardium and visceral pericardium) and intramyocardially, termed as cardiac steatosis [5,6]. Physiologically, epicardial adipose tissue (EAT) has a cardioprotective role on the heart, regulating levels of pro-inflammatory cytokines, stimulating the production of nitric oxide, reducing oxidative stress and protecting coronary arteries against mechanical strain [1,3]. It is currently accepted that myocardial triglyceride accumulation is probably inert [7]. Myocardial energy demands are mainly covered by the oxidation of circulating plasma free fatty acids [8]. However, when excessive free fatty acid delivery is present, such as in obesity or insulin-resistant states, the process exceeds the myocardial oxidative capacity, resulting in myocardial lipid overstorage and lipotoxicity that increase production of reactive oxygen species and cause apoptosis [9]. 

Currently, it is supported that an increase in EAT volume occurs in response to chronic metabolic challenges of the heart, resulting in cytokine upregulation and increased fatty acid oxidation [10]. Ectopic EAT has been suggested to play a significant role in promoting coronary artery atherosclerosis, arrhythmogenesis and heart failure (HF), through dysregulation of various types of adipokines (adiponectin, leptin, tumor necrosis factor alpha (TNF-a), interleukin 6 (IL-6), monocyte chemoattractant protein-1 (MCP-1)) and via increased exosomal miRNAs synthesis [11]. Given that EAT surrounds the myocardium and coronary arteries in the absence of a separating fascia, the ectopic EAT-driven proinflammatory and profibrotic cytokines may diffuse to underlying tissues in a paracrine-dependent manner, contributing to a low grade inflammatory and profibrotic state in the myocardium and vasculature [5]. A heightened state of inflammation in pericoronary adipocytes has been demonstrated in many studies [12]. 

Imaging modalities, such as echocardiography but mostly computed tomography (CT) and cardiac magnetic resonance (CMR) are widely used for detailed fat visualization of cardiac fat deposits. The aim of this review is to provide an overview of the possible additive utility of imaging modalities in screening cardiac adiposity and predicting future arrhythmic risk in patients with increased cardiac fatty depots, along with discussion of underlying disease mechanisms.

## 2. Non-Invasive Imaging Assessment of Cardiac Fat

EAT/PAT volume and thickness can be assessed non-invasively by echocardiography, multidetector CT and CMR, with the two latter able to provide a three-dimensional volumetric quantification (Table 1) [13]. Although EAT and PAT have distinct embryological characteristics, and probably differential clinical effects, there is heterogeneity in the nomenclature used among imaging studies regarding the subgroups of cardiac fat depots, with the term pericardial adipose tissue (PAT) often used to refer to all adipose tissue located epicardially and paracardially (superficial to the pericardium) [14]. 

**Figure 1:**
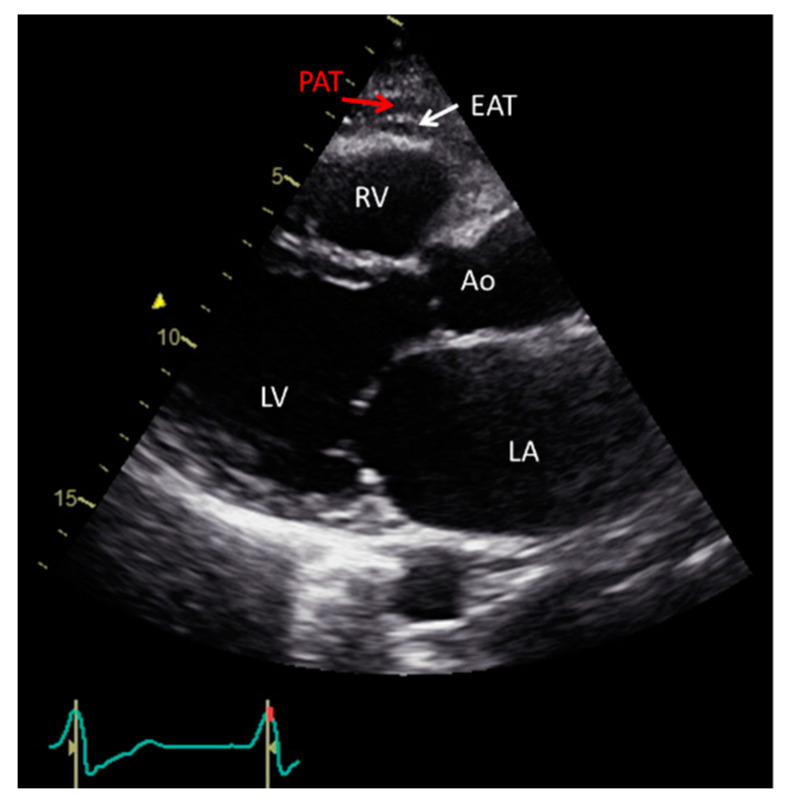
Transthoracic echocardiographic view showing EAT and PAT as echo-lucent areas in front of the RV free wall. EAT is pointed by a white arrow and PAT by a red arrow. Ao: aorta; EAT: epicardial adipose tissue; LA: left atrium; LV: left ventricle; PAT: pericardial tissue; RV: right ventricle.

Echocardiography, is a safe, easily reproducible method, which can measure fat thickness in front of the free right ventricle wall, in the parasternal long and short axis views (Figure 1) [14]. Difficulties in calculating the whole EAT volume and distinguishing the EAT from PAT or pericardial effusion, are the main disadvantages of the method. Α cut-off value >5 mm for EAT thickness has been correlated with increased CV risk [34,35,36,37].

**Figure 2:**
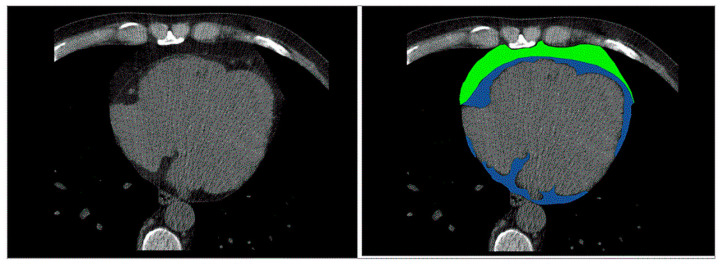
Cardiac Computed Tomography: EAT (depicted in blue) is located between the myocardium and visceral pericardium, PAT (depicted in green) is located adherent and external to the parietal pericardium. EAT: epicardial adipose tissue; PAT: pericardial tissue. de Wit-Verheggen VHW, et al. Cardiovasc Diabetol. 2020;19:129, under Creative Commons license 4.0.

Cardiac CT has been increasingly used for assessment of EAT/PAT (Figure 2) [15]. A radiodensity threshold, of −190 to −30 Hounsfield (HU) units on non-contrast scans and −190 to −3 HU on contrast enhanced CT scans is accurate and reproducible for diagnosis and quantification of EAT volume [16]. In addition to EAT volume, quantification of CT-derived fat attenuation has been correlated with local and systemic inflammatory markers, reflecting unfavorable metabolic activity [17]. In the presence of increased inflammation, higher CT attenuation of EAT is expected. Furthermore, CT can provide information about inflammation of EAT tissue in conjunction with positron emission tomography (PET) [18]. Concurrently, CT provides information about calcification of the coronary arteries and coronary stenoses while its main disadvantage is the exposure to ionizing radiation and nephrotoxicity induced from the contrast material [19,20]. Furthermore, CT can evaluate arterial inflammation in combination with positron emission tomography (PET/CT). In two population-based studies using CT, the Framingham Heart study and the Multi-Ethnic Study of Atherosclerosis, EAT/PAT has been identified as an independent risk predictor for CV disease in the general population [21,22,23]. In keeping with these results, other studies demonstrated that CT-derived EAT/PAT was significantly correlated with high atherosclerotic burden of underlying coronary arteries, incident myocardial infarction and atrial fibrillation (AF) development [24,25,38].

**Figure 3:**
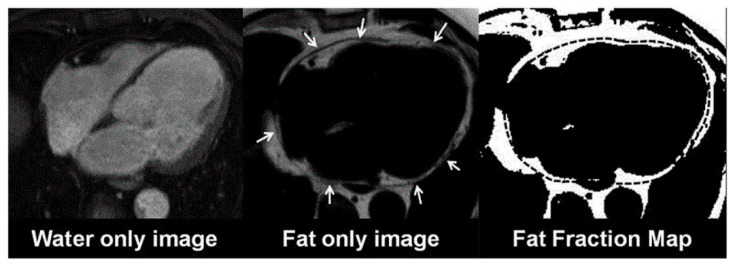
CMR Dixon images. A: Fat only image. B: Fat only Image with the epicardial outlines (arrows). C: Segmented fat voxels with the transferred region of interest. CM: cardiac magnetic resonance. Kropidlowski C, et al. Int J Cardiol Heart Vasc. 2020;27:100477, under Creative Commons license 4.0.

CMR is a noninvasive imaging modality without radiation, able to provide biventricular function assessment, and both tissue characterization and highly reproducible, three-dimensional EAT measurements [26,27]. Assessment of EAT volume does not require the use of gadolinium-based contrast agents and is usually quantified by cine bright-blood steady-state free-precession (SSFP) sequences. Currently, hydrogen proton (1 H) magnetic resonance spectroscopy (MRS) is considered the clinical reference standard for quantifying myocardial triglyceride content, without the need for contrast agents or radionuclides [28]. Spectroscopy can distinguish between multiple myocardial triglycerides, water and creatine based on their different resonance frequencies during 1H-MRS [29]. The spectroscopic volume of interest is usually positioned within the interventricular septum and the spectroscopic signals are acquired with cardiac triggering at end systole. Myocardial steatosis is quantified as the myocardial triglyceride content relative to water or creatine. In addition, newer CMR techniques such as multiecho Dixon-like methods that rapidly obtain fat and water separated images from the region of interest, in a single breath-hold, avoiding contamination from EAT, are also useful tools for this purpose [28,30]. Using the in-phase/out-of-phase cycling of fat and water, water only and fat only images can be created (Figure 3) [30]. This method can also be combined with a variety of sequence types (spin echo, gradient echo, SSFP sequences) and weightings (T1, T2 and proton density). Myocardial fatty infiltration has been linked with diastolic dysfunction, dilated cardiomyopathy and arrhythmogenic right ventricle cardiomyopathy (ARVC) [26,31,32]. Concurrently, EAT/PAT, as assessed by CMR, has been associated with the extent and severity of coronary atherosclerosis, impaired left ventricle (LV) systolic function and myocardial fibrosis in CMR studies [28,33].

The advantages, limitations and clinical implications of different screening modalities in imaging cardiac adiposity are summarized in Table 1. 

## 3. Pathophysiological Mechanisms of AF

AF is the most common clinically relevant arrhythmia. The mechanisms of AF are complex and multifactorial, involving an interaction between initiating triggers, an abnormal atrial substrate and a modulator such as a vagal or sympathetic stimulation [39,40,41]. The triggering of premature atrial contractions by beats that arise especially from one or more pulmonary veins and less frequently from other parts of the atria, may initiate AF while the repetitive firing of these focal triggers may contribute to the perpetuation of the arrhythmia [41,42]. The PVs play an important role in the arrhythmogenesis of AF through the mechanism of automaticity, triggered activity and reentry. Once the arrhythmia has been triggered, different theories, including the multiple wavelet hypothesis and rotors model, have been suggested to explain the maintenance of AF [43,44]. In the first theory, multiple wavelets randomly propagate through the atrial tissue in different directions, detected as complex fractionated electrograms by mapping catheters. In the second theory, the AF is contributed to reentrant electrical rotors, which are identified as wavelets with rotational activity around a structural or functional center detected by spectral analysis of high-frequency sites via intracardiac mapping catheters. 

Increasing evidence supports the role of the autonomic nervous system in the initiation and maintenance of AF through the ganglionic plexuses commonly located on the left atrium in close proximity with epicardial fat pads [45]. Both parasympathetic and sympathetic stimulation enhance propensity to AF, the first by shortening the effective refractory period, whereas the second facilitating induction of AF and automaticity in focal discharge. The role of ablation of ganglionated plexi as an adjunctive procedure in the treatment of AF remains to be determined [46]. 

The development of AF induces a slow but progressive process of atrial substrate abnormalities involving electrical and structural alterations [47]. These changes facilitate electrical reentrant circuits or triggers, which, in turn, increase the propensity for the development and maintenance of the arrhythmia. Electrical remodeling includes shortening of the atrial action potential duration and increased dispersion of refractoriness largely due to downregulation of the l-type Ca^2+^ inward current and upregulation of inward rectifier K+ currents, while heterogeneity in the distribution of intercellular gap junction proteins such a connexin 40 or 43 has been linked with slower conduction velocity, which favors reentry [39,48,49,50,51]. Over time, the presence of AF also leads to structural changes including, hypocontractility, fatty infiltration, inflammation, atrial dilatation and stretch-induced atrial fibrosis which is the hallmark of structural remodeling of AF and is considered especially important substrate for AF perpetuation [52,53,54]. 

Experimental and clinical data indicate that inflammation is particularly involved in the initiation and maintenance of AF and conversely AF can further promote inflammation [55,56]. Although, the precise mechanistic links remain unclear, several effects of inflammation seem to be mediated by oxidative stress [57]. Various inflammatory biomarkers including C-reactive protein (CRP), IL-6, TNF-α, and MCP-1 are associated with AF risk [58,59]. It has been suggested that TNF-α, IL-2 and platelet-derived growth factor can provoke abnormal triggering in PVs and shortening of atrial action potential duration through regulation of calcium homeostasis, as well as induce atrial fibrosis, connexin dysregulation and apoptosis leading to increased conduction heterogeneity [55]. However, their clinical utility in guiding AF management is not well established [56,58].

## 4. Cardiac Adiposity and AF 

Even though cardiac fat depots encompass a small minority of total body fat, their proximity with cardiac structures has raised great interest whether they can play an additional role in the modulation of biochemical and metabolic triggers leading to AF. Increasing evidence supports a potential role of EAT/PAT as a determinant of the substrate of AF as well as a modulator and/or trigger (Table 2) [60]. Furthermore, fatty infiltrates provide a substrate (class IVf) for arrhythmia genesis according to European Heart Rhythm Association consensus [61]. The underlying mechanism linking EAT/PAT and AF appears to be multifactorial with local inflammation, fibrosis, adipocyte infiltration, electrical remodeling, autonomic nervous system modulation, oxidative stress and gene expression playing interrelating roles [62].

Cardiac imaging modalities have demonstrated a strong direct relation between cardiac adiposity and AF pathogenesis. A significant relation between PAT, quantified by CT, and the development of AF was reported in the Framingham Heart Study enrolling over 3000 patients, after adjustment for AF risk factors, including BMI [63]. EAT/PAT has also been associated with AF severity and left atrial volume, and was an adverse prognostic marker for AF recurrence after catheter ablation, as determined by various imaging modalities including CMR and echocardiography [64,65,66,67,68,80,81,83,87]. Specifically, in studies using CT EAT/PAT volume was larger in AF patients and was independently associated with paroxysmal and persistent AF, while EAT volume and thickness of periatrial EAT were related to the chronicity of AF [64,69]. Consistently, periatrial EAT volume was a predictor of new-onset AF in patients with CAD and postoperative AF in patients undergoing coronary artery bypass grafting [70,71]. EAT volume has been associated with negative ablation outcomes, although this was not confirmed in a very recent hybrid AF ablation study, signifying that further research is required to clarify the effect of EAT on these procedures [88]. Additionally, EAT thickness, as assessed by echocardiography, was useful in predicting adverse CV events, and could provide incremental value for CV outcome prediction over traditional clinical and echocardiographic parameters in AF [84].

There is increasing evidence supporting a close association between EAT/PAT and inflammation in CT-derived studies. Thus, inflammatory activity of EAT, reflected by glucose metabolism in PET/CT, was significantly and strongly linked with AF [72]. In line with this, inflammation of local periatrial EAT, as expressed by higher CT-density, was related to the presence of paroxysmal AF compared to controls [73]. Moreover, increased EAT volumes and elevated levels of inflammatory makers, such as CRP and interleukins, were noted in persistent AF rather than paroxysmal AF patients [74]. Additionally, another study showed that samples of pericoronary, periventricular and periatrial EAT, obtained from patients paired for CV risk factors, CAD and AF, appeared to have varying pro-inflammatory properties dependent on its anatomical location, underscoring that imaging assessment of each EAT compartment might add value in the risk of AF and CAD [89]. Finally, given that obesity is a well-established risk factor for AF and is associated with EAT/PAT, together with a growing body of evidence linking inflammation with pathogenesis of AF, indicate a potential interaction between local and systemic inflammation in the increasing prevalence of AF [90,91,92,93].

Moreover, cardiac adiposity can play a role on atrial electrophysiology, promoting functional heterogeneity, which contributes to conduction abnormalities. Complex fractionated atrial electrograms and high dominant frequency sites, both playing an important role in the maintenance of AF, were close related with CT-derived EAT/PAT volume and locations which are frequent targets for AF catheter ablation [75,76]. This correlates with the fact that the presence of EAT/PAT in CT or CMR was linked with alterations in atrial conduction, such as slower conduction velocity, prolonged cardiomyocyte field potential duration, greater complexity of activation patterns, lower bipolar voltage and electrogram fractionation [77,78,79,82].

Additionally, EAT/PAT may affect arrhythmogenesis by triggering sympathetic tone through the adrenergic and cholinergic nerves it contains, and by promoting fibrosis, which play a central role in the AF-pathophysiology, via cytokines and growth factors secretion [94]. Of note, in patients with and without heart failure echocardiographic EAT thickness was related to sympathetic nervous system imbalance, as detected by myocardial scintigraphy, impaired heart rate variability and heart rate turbulence parameters [85,86]. Recently, additional insights into the impact of EAT on the atrial substrate for AF have emerged from the correlation of local CT-EAT volume with histological atrial fibrosis, an effect that can be attributed to an EAT-cardiomyocyte paracrine axis [77]. Finally, intramyocardial fat has also been associated with supraventricular arrhythmias. Fatty infiltrates, which are common atrial histological findings, may become fibrotic under specific disease conditions, affecting the myocardial remodeling processes involved [95]. Fibro-fatty infiltration of the subepicardium has been recognized as an important determinant of the substrate of AF [96,97]. 

## 5. Cardiac Adiposity and Ventricular Arrhythmias 

Although the link of EAT/PAT with AF is strong, its relation with ventricular arrhythmias currently remains insufficiently validated. In contrast, there is association between fatty infiltration of the myocardium and cardiomyopathies. Usually this subset of patients have significant local and diffuse fibrosis, proinflammatory states, and comorbidities that predispose them to arrhythmias. Intramyocardial fat has been connected with ventricular arrhythmogenesis in obese adults, genetic disorders, such as arrhythmogenic right ventricular cardiomyopathy, myotonic dystrophy, Fabry’s disease, as well as healed myocardial infarction and systolic heart failure (Table 3) [98,99].

Reentry is the responsible mechanism for most ventricular arrhythmias, while focal mechanism, probably through triggered activity arising from either early or delayed afterdepolarizations without evidence of reentry, may also contribute to ventricular arrhythmias [121,122]. Multiple factors including underlying structural myocardial disease, mechanical factors such as increased wall stress and LV dilation, neurohormonal factors via sympathetic nervous and renin–angiotensin systems activation as well as myocardial ischeamia lead to alteration of electrophysiogical milieu, including changes in conduction and refractoriness and enhanced automaticity. 

### 5.1. Arrhythmogenic Right Ventricular Cardiomyopathy (ARVC)

ARVC is a hereditary cardiomyopathy, characterized by fibrofatty replacement of the ventricular myocardium, with the right ventricle (RV) being predominantly affected, although left or biventricular forms have been also described [123]. The altered histopathological substrate predisposes these patients to ventricular arrhythmias and sudden cardiac death. CMR is considered the preferred imaging modality, being able not only to quantify biventricular function, but more importantly to assess myocardial tissue abnormalities, such as intramyocardial fat infiltration, oedema and fibrosis. Although fibrosis and/or fibrofatty replacement of myocytes by LGE is the pathologic hallmark of ARVC, these findings are not included in the 2010 revised Task Force Criteria (TFC) for the diagnosis of ARVC, because of concerns about their subjectivity, specificity and reproducibility [124].

Even though the direct assessment of RV tissue composition by CMR is challenging, technical advances in imaging, such as the cine-SSFP techniques, may provide better characterization of fatty content and contribute to a better stratification of arrhythmic risk in ARVC patients [123]. Thus, fatty infiltration was associated with advanced RV structural disease in patients that fulfilled major TFC-CMR imaging criteria and who were at the highest arrhythmic risk [103]. Of note, cardiac steatosis was also found in a minority of patients with partial TFC imaging criteria, suggesting a potential role for diagnosis and reclassification of patients who would otherwise not meet current CMR imaging criteria. These findings were further expanded when the involvement of left ventricle (LV) was considered in this disease setting. Recently, LV intramyocardial fat was detected in more than half of ARVC patients, was mostly located in the same regions of fibrotic deposition and was negatively related with the severity of LV systolic impairment [104,105]. Concomitantly, LV fat infiltration in combination with LV wall motion abnormalities and LGE could independently predict the major combined endpoint of sudden cardiac death, aborted cardiac arrest, and appropriate cardioverter-defibrillator implantation in ARVC patients [106,107]. LV involvement also allowed a reclassification of 5-year risk of events compared with the ARVC score. The above mentioned studies highlight the need for further research to examine the potential additive utility of adiposity and/or fibrosis in ARVC patients that are in an early stage of the cardiomyopathy. 

In addition, CT has also been used for depiction of fatty infiltration within the thin RV wall, due to its high spatial resolution, combined with the high native contrast of adipose tissue [123]. Intramyocardial fat burden was correlated with RV dysfunction and VT substrate, such as conduction and repolarization disturbances, in ARVC [100,101]. A vast majority of the local abnormal ventricular activities were located around the border of the RV fat segmentation, indicating that the interrogation of CT with 3-dimensional electroanatomic mapping could demonstrate ablation targets. Finally, EAT was an indicator of the degree of myocardial disease progression in ARVC, since it was related to the severity of structural disease in the RV [102]. 

### 5.2. Healed Myocardial Infarction 

Histological and imaging studies have revealed that intramyocardial fat deposition is located frequently in post-infarcted ventricular myocardium during a healing process called lipomatus metaplasia [125,126,127]. An association between lipomatus metaplasia and abnormal ventricular electrophysiology has been reported in both animal and clinical studies [110,128]. In this regard, electrophysiological studies demonstrated that lipomatus metaplasia, as depicted by CMR or CT, was strongly associated with scar age and size, lower bipolar and unipolar amplitudes and critical ventricular tachycardia circuit sites in patients with ischemic cardiomyopathy, suggesting its potential role in the generation of scar-related VT circuits in this setting [108,109]. Fragmented and isolated electrograms were also more frequently observed in areas with fat. Importantly, intramyocardial adipose, predominantly detectable within the subendocardial layer of scar area with variable transmural extent, was a significant predictor of sustained ventricular arrhythmia, heart failure hospitalizations and all-cause mortality in patients with history of myocardial infarction [110]. These results expand the findings of histological studies where intramyocardial adiposity was associated with significantly altered ventricular electrophysiology and increased propensity for VT after MI, whereas there was an inverse link with myocardial viability [128,129]. Myocardial fat was associated with altered electrophysiological properties and VT circuit sites in patients with ICM.

Recently, it has become evident that EAT, as documented using CMR or CT, was an independent predictor of VT recurrence and all-cause mortality following ablation, highlighting the role of this imaging biomarker for risk stratification post-ablation [111]. Consistently, the different electrophysiological properties of VT substrate according to the presence of fat was also confirmed.

### 5.3. Heart Failure (HF) and Other Conditions

EAT/PAT is increased in patients with LV hypertrophy, diastolic dysfunction, and heart failure with mid-range and preserved ejection fraction, whereas regression of EAT has been reported in advanced heart failure [130,131,132,133,134]. However, the presence of EAT/PAT seems to be associated with ventricular arrhythmias in the setting of heart failure with reduced ejection fraction. Thus, CMR-derived PAT was related with the development of ventricular tachycardia/fibrillation and mortality in patients with systolic HF [112]. In line with this, recently, echocardiographic assessment of EAT was a strong predictor of both clinical and arrhythmic events, including ventricular tachycardia/fibrillation and AF [114]. Furthermore, intarmyocardial fat was significantly related to LV global function and fibrosis volume in patients with dilated cardiomyopathy, indicating that it may be a stronger marker of disease prognosis [113].

Moreover, EAT/PAT was independently associated with prolonged QTc interval and frequent ventricular premature beats in different subgroups of patients, indicating the arrhythmogenic potential of cardiac adiposity [115,116,117,118]. Additionally, EAT was an independent marker of impaired heart rate recovery, a noninvasive index of autonomic nerve dysfunction in obese patients with obstructive sleep apnea, portending poor cardiovascular prognosis in obese patients [119]. Finally, echocardiography-derived EAT thickness was higher in patients with premature ventricular contraction ablation failure [120].

## 6. EAT/PAT as a Therapeutic Target

Given its relation to metabolic dysregulation, inflammation, free fatty acid delivery and glucose resistance, EAT/PAT has become a therapeutic target for life style modifications and pharmacological therapies modulating fat, as well as those improving glucose control. Emerging evidence shows that EAT may be reduced by diet, exercise, bariatric surgery, statins and antidiabetic therapies including, glucagon-like peptide-1 (GLP-1) analogues and sodium-glucose co-transporter inhibitors (SGLT2is) [135,136,137,138,139,140,141,142,143,144,145,146,147,148,149,150,151,152,153,154,155]. However, it is not known yet whether a reduction in EAT volume can be translated into clinically relevant reduction in cardiovascular risk.

In particular, recent studies have shown that exercise training may be a means to specifically target cardiac adipose tissue, as exercise led to a reduction in EAT/PAT volume ranging from 5% to 32%, even in the absence of weight loss [135,136,137,138]. Accordingly, significant reductions in both EAT/PAT volume and total cardiac adipose tissue volume have been reported following dietary restrictions and bariatric surgery [139,140,141]. Nevertheless, given that the two latter modalities compared with exercise have larger effects on body loss than on VAT reduction in obese people, it is likely that they are not optimal to target EAT [142].

In regard to pharmaceutical interventions, significant reductions in EAT/PAT volume was found following administration of atorvastatin in patients with AF, while statin therapy significantly reduced both EAT thickness and its inflammatory status in fat samples obtained from patients undergoing cardiac surgery [143,144,145]. Furthermore, liraglutide, a GLP-1 analogue that has been shown to reduce CV mortality, caused an almost 40% reduction in EAT/PAT among type 2 diabetic patients, underscoring that the cardioprotective effects of this drug could be potentially mediated through the reductions in EAT [146]. Accordingly, SGLT2is prevent CV deaths and HF events regardless of the presence or absence of diabetes [147]. It remains unknown how SGLT2is exert such beneficial effects on CV diseases, since SGLT2 is not expressed in cardiomyocytes [148]. A theory is that SGLT2is have a salutary effect through increased lipolysis in adipose tissue by reducing plasma glucose levels, leading to increased free fatty acids delivery to the heart while reducing the EAT depot [136,148]. The effect of SGLT2is on EAT/PAT has been investigated only recently. Thus, EAT thickness and/or volume was significantly decreased by dapagliflozin, canagliflozin, ipragliflozin and luseogliflozin, suggesting a drug class effect [149,150,151,152,153,154,155]. Of note, recent studies have reported that dapagliflozin (a) improved the differentiation of epicardial adipocytes, (b) benefited wound healing in endothelial cells, (c) reduced EAT volume, (d) decreased secretion of proinflammatory chemokines and e) of P-wave indices, such as P-wave dispersion [150,151]. The changes in P-wave indices were especially associated with changes in EAT volume [150].

Although, EAT/PAT shows promise as a modifiable cardiac risk factor, there are still several aspects to be clarified and more tailored therapeutic strategies, related to inflammation and metabolic dysfunction, to be investigated, before we understand whether EAT will guide future clinical decision-making.

## 7. Future Perspectives

Current imaging modalities have provided valuable insight into the relationships between cardiac adiposity and arrhythmogenesis, in order to better understand the pathophysiology and improve risk prediction and re-stratification, over and above the presence of obesity and traditional risk factors, especially in patients who are considered to be at intermediate risk. However, at present, given the insufficient data for the additive value of imaging biomarkers on commonly used risk algorithms, the use of different screening modalities currently is indicated for personalized risk stratification and prognostication in this setting. Furthermore, a qualitative evaluation of adipose tissue next to its quantification may be more clinically relevant. Thus, the evaluation of cardiac metabolism and detection of tissue inflammation by newer imaging methods, such as 31-phosphate MRS, hyperpolarized 13C MRS and CT-derived fat attenuation index, may give more information for the arrhythmogenic substrate at an early stage [156,157,158]. Moreover, the application of PET, using a variety of tracers that can quantify fatty acid, oxygen, glucose, and lactate uptake, may further stimulate research for the evaluation of cardiac metabolism in arrhythmia genesis [159].

Imaging biomarkers may also guide therapeutic strategies targeting cardiac fat depots and monitor responses to treatment [136]. Nevertheless, it is not yet known whether reducing cardiac fatty depots will also differentiate the arrhythmogenic substrate and reduce the risk of developing arrhythmia.

## 8. Conclusions

Although there is extensive experimental, imaging and clinical evidence that cardiac adiposity is an important modulator of arrhythmogenicity, mainly of AF, several aspects need clarification. Variable strengths of causative relationship have been suggested by many screening studies including different populations, different disease stages and different fat locations (periatrial, periventricular, perivascular) and indexes (volume, thickness). In addition, EAT and PAT are often not discriminated on screening modalities. Moreover, a standardized imaging measurement protocol and threshold values for different subgroups with comorbidities (hypertension, diabetes, obstructive sleep apnoea) are still lacking. Future research will enhance our understanding about the diagnostic and prognostic significance of multimodality imaging of cardiac adiposity as a marker of arrhythmias and whether it may contribute to the management of at-risk or affected patients.

## Figures and Tables

**Table 1:** Cardiac adiposity screening by imaging modalities. Advantages, limitations and clinical implications apart from arrhythmias.

	CT [15,16,17,18,19,20,21,22,23,24]	CMR [25,26,27,28,29,30,31,32,33]	Echocardiograhy [14,34,35,36,37]
Advantages	• EAT/PAT assessment: - volumetric technique - 3-dimensional EAT measurement - high reproducibility - better spatial resolution than CMR - EAT assessment on contrast and non-contrast scans • Additional information: - relation of EAT radiodensity with metabolic processes - calcification of the coronary arteries - coronary artery stenosis - anatomical and metabolic data with PET/CT	• EAT/PAT assessment: - volumetric technique - 3-dimensional EAT measurement - high reproducibility - no radiation exposure - no use of contrast agents • Myocardial fatty infiltration assessment by: - 1H-MRS - multiecho Dixon methods • Additional information: - biventricular function assessment - LV mass - LA volume - fibrosis by LGE	• EAT/PAT thickness assessment: - relatively inexpensive - widely available - no radiation exposure • Additional information: - biventricular function assessment - LV mass - LA volume
Limitations	- radiation exposure - nephrotoxicity	• CMR: - lack of availability/expertise - high cost - marked obesity - claustrophobia - often the pericardium not clearly seen on inferior slices of CMR scans - impossible to scan CMR-unsafe devices (metallic clips, pacemakers, defibrillators) • 1H-MRS - lack of availability/expertise - high cost - contamination from EAT/PΑΤ	- no volumetric EAT estimation - difficulties in distinguishing the EAT from PAT or pericardial effusion - dependent on operator’s - experience
Clinical implications	• EAT/PAT is associated with - adverse CV outcome - CAD - coronary artery calcification	• EAT/PAT is associated with - presence/severity of CAD - impaired LV systolic function - myocardial fibrosis • Myocardial fatty infiltration associations - diastolic dysfunction - dilated cardiomyopathy - ARVC - myocardial fibrosis	• EAT thickness is associated with: - presence/severity of CAD - LV hypertrophy - diastolic dysfunction - HFpEF/HFmrEF - metabolic syndrome - carotid atherosclerosis - Framingham risk score

Abbreviations: ARVC: arrhythmogenic right ventricle cardiomyopathy; CAD: coronary artery disease; CMR: cardiovascular magnetic resonance; CT: computed tomography; CV: cardiovascular; EAT: epicardial adipose tissue; 1H-MRS: hydrogen proton magnetic resonance spectroscopy; HFmrEF: heart failure with mid-range ejection fraction; HFpEF: heart failure with preserved ejection fraction; LA: left atrium; LGE: late gadolinium enhancement; LV: left ventricle; PAT: pericardial adipose tissue; PET: positron emission tomography.

**Table 2:** Relationship between imaging measures of cardiac adiposity and AF.

CT	CMR	Echocardiography
EAT/PAT is associated with: • Histological atrial fibrosis • Development of AF - volume/thickness is correlated with: paroxysmal AF persistent AF post-CABG AF ablation failure [63,64,65,66,67,68,69,70,71] • Inflammation - SUV in LA-EAT by PET/CT - volume/thickness is correlated with CRP and IL-6 in persistent AF - density is correlated with paroxysmal AF [72,73,74] • Atrial electrophysiology - fractionated atrial electrogram - high dominant frequency sites - slow atrial conduction velocity - prolonged potential duration - lower bipolar voltage - targets for AF catheter ablation [75,76,77,78,79]	EAT/PAT is associated with: • Development of AF - volume/thickness is correlated with: severity of AF LA volumes ablation failure [80,81] • Atrial electrophysiology - low atrial voltage - fractionated signals - LA conduction abnormalities [82]	EAT/PAT thickness is associated with: - ablations failure - adverse CV events [83,84] • Sympathetic nervous system imbalance - impaired heart rate variability - impaired heart rate turbulence parameters - correlation with cardiac ^123^ I-MIBG planar and SPECT parameters [85,86]

Abbreviations: AF: atrial fibrillation; CABG: coronary artery bypass grafting; CMR: cardiovascular magnetic resonance; CRP: C-reactive protein; CT: computed tomography; CV: cardiovascular; EAT: epicardial adipose tissue; IL-6: interleukin 6; LA: left atrium; MIBG: metaiodobenzylguanidine; PAT: pericardial adipose tissue; PET: positron emission tomography; SPECT: single-photon emission computed tomography; SUV: standardized uptake value.

**Table 3:** Relationship between imaging measures of Cardiac Adiposity and Ventricular Arrhythmias.

	CT	CMR	Echocardiography
ARVC	Myocardial fat infiltration is associated with: - RV dysfunction - VT substrate (conduction and repolarization disturbances) [100,101,102]	Myocardial fat infiltration is associated with: • Diagnosis of ARVC - severity of RV structural disease - impaired RV functional status - impaired LV systolic function [103,104,105] • Stratification of ARVC-patients LV fat infiltration: - is a predictor of VT/VF, SCD and aborted cardiac arrest - allows a reclassification of 5-year risk of events compared with the ARVC score [106,107]	
Healed myocardial infarction	Myocardial fat infiltration is associated with: - scar age and size, - lower bipolar and unipolar amplitudes - fragmented electrograms - colocalization with critical VT isthmuses - adverse outcomes including postablation VT recurrence and all-cause mortality [108,109]	• Myocardial fat infiltration is associated with: - larger infarcts - adverse LV remodeling - sustained VT, HF hospitalization and all-cause mortality [110] • PAT is associated with: - postablation VT recurrence [111]	
HF		• PAT is associated with: - development of VT/VF and - mortality in patients with systolic HF [112] • Myocardial fat infiltration is related with: - LV global function and - fibrosis volume, in patients with DCM [113]	EAT thickness is a predictor of: - clinical events and - arrhythmic events (VT/VF and AF) [114]
Other conditions	RV-PAT is associated with - the frequency of VPBs [115]		EAT thickness is associated with: - prolonged QTc interval in hypertensive pts and in general population - the frequency of VPBs in pts - Without structural heart disease - impaired post-exercise HRR in obese pts with obstructive sleep apnea - VPBs ablation failure - [116,117,118,119,120]

Abbreviations: AF: atrial fibrillation; ARVC: arrhythmogenic right ventricle cardiomyopathy; CMR: cardiovascular magnetic resonance; CT: computed tomography; DCM: dilated cardiomyopathy; EAT: epicardial adipose tissue; HF: heart failure; HRR: heart rate recovery; LV: left ventricle; PAT: pericardial adipose tissue; RV: right ventricle; SCD: sudden cardiac death; VF: ventricular fibrillation; VPBs: ventricular premature beats; VT: ventricular tachycardia.

## Data Availability

Not applicable.
